# Development of multiple strain competitive index assays for *Listeria monocytogenes *using pIMC; a new site-specific integrative vector

**DOI:** 10.1186/1471-2180-8-96

**Published:** 2008-06-13

**Authors:** Ian R Monk, Pat G Casey, Michael Cronin, Cormac GM Gahan, Colin Hill

**Affiliations:** 1 Department of Microbiology, University College Cork, Cork, Ireland; 2 Alimentary Pharmabiotic Centre, University College Cork, Cork, Ireland; 3 Department of Maths and Statistics, University College Cork, Cork, Ireland; 4 Department of Pharmacy, University College Cork, Cork, Ireland

## Abstract

**Background:**

The foodborne, gram-positive pathogen, *Listeria monocytogenes*, is capable of causing lethal infections in compromised individuals. In the post genomic era of *L. monocytogenes *research, techniques are required to identify and validate genes involved in the pathogenicity and environmental biology of the organism. The aim here was to develop a widely applicable method to tag *L. monocytogenes *strains, with a particular emphasis on the development of multiple strain competitive index assays.

**Results:**

We have constructed a new site-specific integrative vector, pIMC, based on pPL2, for the selection of *L. monocytogenes *from complex samples. The pIMC vector was further modified through the incorporation of IPTG inducible markers (antibiotic and phenotypic) to produce a suite of four vectors which allowed the discrimination of multiple strains from a single sample. We were able to perform murine infection studies with up to four EGDe isolates within a single mouse and showed that the tags did not impact upon growth rate or virulence. The system also allowed the identification of subtle differences in virulence between strains of *L. monocytogenes *commonly used in laboratory studies.

**Conclusion:**

This study has developed a competitive index assay that can be broadly applied to all *L. monocytogenes *strains. Improved statistical robustness of the data was observed, resulting in fewer mice being required for virulence assays. The competitive index assays provide a powerful method to analyse the virulence or fitness of *L. monocytogenes *in complex biological samples.

## Background

Historically, the measurement of bacterial virulence has involved the use of infection models to access the ability of a pathogen to cause disease. One widely used method is the LD_50 _assay, which is defined as the number of bacteria required to kill 50% of the infected hosts. This method yields valuable data pertaining to the cumulative, absolute virulence of the bacterium, which can theoretically be compared between laboratories. But as detailed by Beuzón and Holden [1], it is a crude method as it does not relay information on the infection kinetics. Also, if the deletion of a gene does not increase the LD_50_, it does not necessarily mean that the gene product does not play a role in the virulence of the bacterium. If two or more strains (e.g. wild type to mutant or independent isolates) could be compared within a single host, the kinetics of infection could be monitored, which could expose subtle differences. Intra-animal experiments would help to minimise inherent inter-animal biological variation and also improve the identification of mutations or isolates with reduced competitive fitness within the host [2]. This form of assay has been termed a "competitive index" and is becoming an increasing popular method to examine bacterial virulence [3-5]. One crucial factor is to establish the ability to discriminate strains, without adversely impacting on the natural fitness of the organism.

*Listeria monocytogenes *is a foodborne pathogen, which can cause fatal infections in a susceptible host [6]. In humans, the initial step of gastrointestinal invasion is mediated via the interaction of a bacterial cell surface exposed protein, Internalin A (InlA), with the host cell surface ligand, E-cadherin [7]. Subsequent intracellular replication and spread can lead to systemic disease. While the mouse is a poor model of listerial gastrointestinal disease due to the limited affinity of InlA for murine E-cadherin, the tools required for systemic proliferation are functional once the gastrointestinal tract is bypassed via intravenous or intraperitoneal injection [8]. Identification and assessment of genes required for the virulence of *L. monocytogenes *has been greatly helped by the sequencing of multiple genomes [9,10]; however, functional post genomic analysis requires the development of improved techniques for the discrimination of virulence potential. LD_50 _measurements are still widely used, but for the reasons described above, competitive indices could provide a more elegant alternative. Even though competitive index assays have been previously applied to *L. monocytogenes *[11-13], these are not broadly applicable and have inherent limitations (see Discussion section).

Here, we have established a method to stably tag *L. monocytogenes *strains and assess their competitive fitness in complex samples. Through the development of a new site-specific integrative vector, pIMC, we have produced four derivatives with different IPTG inducible antibiotic and phenotypic markers for their subsequent discrimination from complex samples.

## Results

### Construction of pIMC

**Figure 1:**
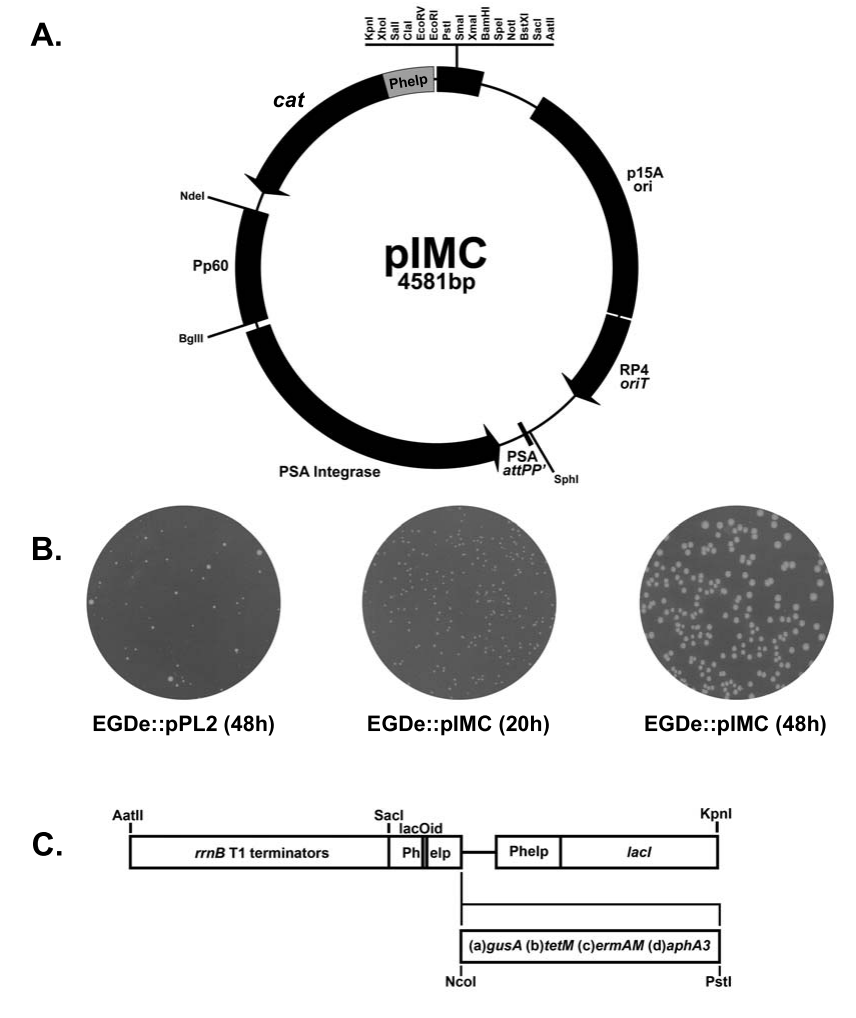
**Plasmid maps for pIMC and IPTG inducible marker expression in pIMC3**. The plasmid (A) pIMC was created by SOE PCR (see Material and Methods) from oligonucleotides described in Table 1. The backbone of pIMC, derived from pPL2 [11] encodes the p15A low copy *E. coli *origin of replication and RP4 conjugative origin of transfer. No gram-positive origin of replication is present on the plasmid, therefore upon transformation, chromosomal integration into *L. monocytogenes *tRNA^ARG ^is directed by the Listeriophage PSA integrase. Antibiotic selection is supplied by the chloramphenicol acetytransferase (*cat*) fused to the highly expressed listerial promoter (Phelp) [13]. Restriction sites labelled on pIMC are unique and for sequencing purposes, T3 and T7 primer binding sites are present before the *Kpn*I and after the *Sac*I restriction sites, respectively. The plasmid sequence is accessible under the EMBL nucleotide accession number AM940001. A comparison of chloramphenicol selection (7.5 μg/ml) is shown in (B), with pIMC exhibiting uniform colony size compared to pPL2 transformed EGDe. (C) Antibiotic markers (kanamycin (*aphA3*), erythromycin (*ermAM*) and tetracycline (*tetM*)) and the beta-glucuronidase marker (*gusA*) were subcloned from pIMK3 into pIMCa as a *Sac*I/*Pst*I fragment (see Materials and Methods).

We have modified the *Listeria *site-specific integrative vector, pPL2 [11], by a series of SOE-PCR reactions to generate pIMC (Fig 1a). The backbone of pPL2 was conserved, including the pBluescriptII KS multiple cloning site, low copy *E. coli *origin of replication, conjugative transfer functions, and the listerial phage integrase genes. To improve antibiotic selection and decrease vector size, the gram-negative *cat *gene was deleted and the inducible gram-positive *cat *promoter [12] replaced with the highly expressed listerial promoter (Phelp) [13]. As shown in Fig 1b, *L. monocytogenes *strain EGDe, when transformed with pPL2, exhibited variable colony size and took 48 h to yield colonies. Even upon passage in the presence of chloramphenicol, EGDe::pPL2 yielded a variable colony size and could not grow at chloramphenicol concentrations above 7.5 μg/ml (data not shown). These results suggested that the inducible gram-positive *cat *gene, as a single copy on the chromosome, was a poor selection marker [12]. In contrast, EGDe transformed with pIMC yielded uniformly sized colonies, which were visible after 20 h (Fig 1b). EGDe::pIMC exhibited similar growth kinetics to EGDe and was able to grow in the presence of up to 75 μg/ml of chloramphenicol. The integration of pIMC in EGDe and two additional *L. monocytogenes *strains (10403S and F2365) was highly stable. After 10 serial passages at 37°C in BHI broth (1:1000 dilution) without antibiotic selection, none of the 100 colonies screened from each strain had lost the plasmid. This vector should prove useful for genetic complementation and as a marker for *in vitro *and *in vivo *strain selection.

### Development of *in vivo *multiple competitive index assay

Comparative analysis of virulence potential is a cornerstone in understanding the ability of a pathogen to cause disease and in determining the relative contribution of putative virulence factors. We applied pIMC to establish a widely applicable method to evaluate virulence potential during mixed infections, in the form of a competitive index (CI) [1]. To exploit the high level of chloramphenicol resistance of pIMC, which allows selection from complex samples, we subcloned three IPTG-inducible antibiotic resistance markers *ermAM *(Ery^R^), *aphA3 *(Kan^R^), *tetM *(Tet^R^) and an IPTG-inducible phenotypic marker, *gusA *(Gus) into pIMCa (see Material and Methods). These markers enable strains to be distinguished directly from each other by antibiotic selection or through X-gluc hydrolysis (leading to a blue colony colour). These plasmids were electroporated into EGDe and the resultant strains were evaluated *in vitro *for growth rate and *in vivo *for virulence. No differences in growth rate were observed between the strains when grown in BHI (data not shown).

**Figure 2:**
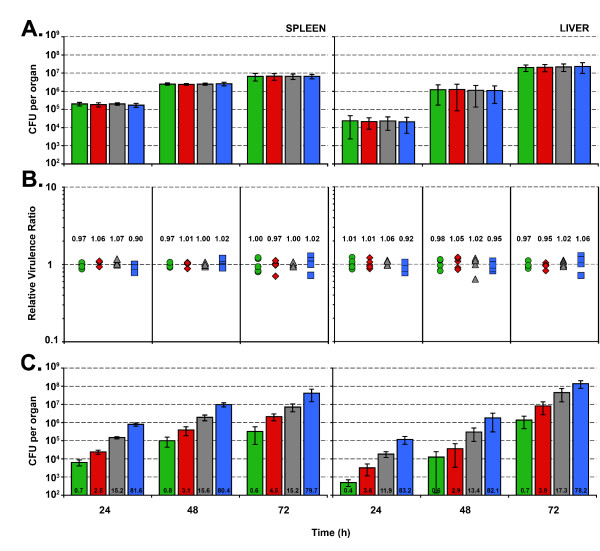
**Growth kinetics within Balb/c mice after quadruple intravenous infection with EGDe::pIMC3 derivatives**. (A) A tail vein injection with *L. monocytogenes *isolate EGDe was administered into 15 Balb/c mice with a total inoculum of 2 × 10^4 ^CFU per 100 μl. The inoculum was composed of an equal ratio of EGDe *wt *(5 × 10^3 ^CFU) tagged with the four different pIMC3 plasmids. On each subsequent day, 5 mice were sacrificed, with the spleens and livers enumerated on different BHI agars as described in the materials and methods. Bars correspond to EGDe; green (::pIMC3*kan*), red (::pIMC3*ery*), grey (::pIMC3*tet*) and blue (::pIMC3*gus*). Data are presented as mean CFU with standard deviation from five organs. The relative virulence ratio (RVR) (B) was calculated from the data presented in (A). Per mouse, the proportion of each strain comprised within the organ (liver or spleen) (output %) was divided by the proportion of the strain in the initial inoculum (input %) and presented as an individual data point (using the formula in additional file 2). The mean RVR was calculated from the average of 5 organs from each strain. A value of 1 indicates no change in the relative ratio. Symbols correspond to EGDe; green circle (::pIMC3*kan*), red diamond (::pIMC3*ery*), grey triangle (::pIMC3*tet*) and blue square (::pIMC3*gus*). (C) A tail vein infection with *L. monocytogenes *isolate EGDe was administered into 15 Balb/c mice with a total inoculum of 2 × 10^4 ^CFU per 100 μl. The inoculum was composed of EGDe *wt *tagged with pIMC*kan *(0.75%), pIMC*ery *(2.75%), pIMC*tet *(13.25%) and pIMC*gus *(83.25%) in a skewed ratio. The number on each bar gives the mean percentage for each strain recovered from the spleen or liver. Bars correspond to EGDe; green (::pIMC3*kan*), red (::pIMC3*ery*), grey (::pIMC3*tet*) and blue (::pIMC3*gus*).

In the initial *in vivo *experiments, the four EGDe tagged strains were mixed at a 1:1:1:1 ratio and then administered by intravenous injection to 15 BALB/c mice. On each subsequent day, 5 mice were sacrificed, with the bacterial loads in the spleen and liver enumerated. Total counts recovered from the spleen and liver throughout a three day time course were consistent with a typical EGDe infection (Fig 2a and data not shown), suggesting the tagging and marker expression do not impact on the virulence of EGDe. We were able to distinguish the four tagged strains with a double selection, using both chloramphenicol and an additional marker for selection or discrimination. We found that chloramphenicol at a concentration of 7.5 μg/ml was sufficient to remove background host bacterial flora on BHI agar from mouse faecal and intestinal samples. Counts from both organs indicated that each strain maintained a competitive index close to 1.0 (Fig 2b), thereby maintaining the original inoculum ratio within each mouse. This demonstrates that none of the four markers had an adverse impact on virulence. The kinetics of infection through the IV route revealed that the spleen was more permissive to initial infection than the liver, consistent with the results of other researchers [14]. In a second experiment, we tested whether the ratios would be maintained throughout an infection if the composition of the initial inoculum was skewed (83.3(*gus*):13.2(*tet*):2.75(ery):0.75(*kan*)). As before, ratios were maintained throughout the course of the infection (Fig 2c). The Gus tagged strain is discriminated by a colourimetric change, which is not directly selective and thus may be overlooked at very low recovery rates (e.g. if virulence has been dramatically affected). The other three markers have the advantage of clean selection; however if four strains are required, we recommend that the dominant strain (e.g. the wild type) should be tagged with this marker.

### Virulence comparison of different *L. monocytogenes *strains

**Figure 3:**
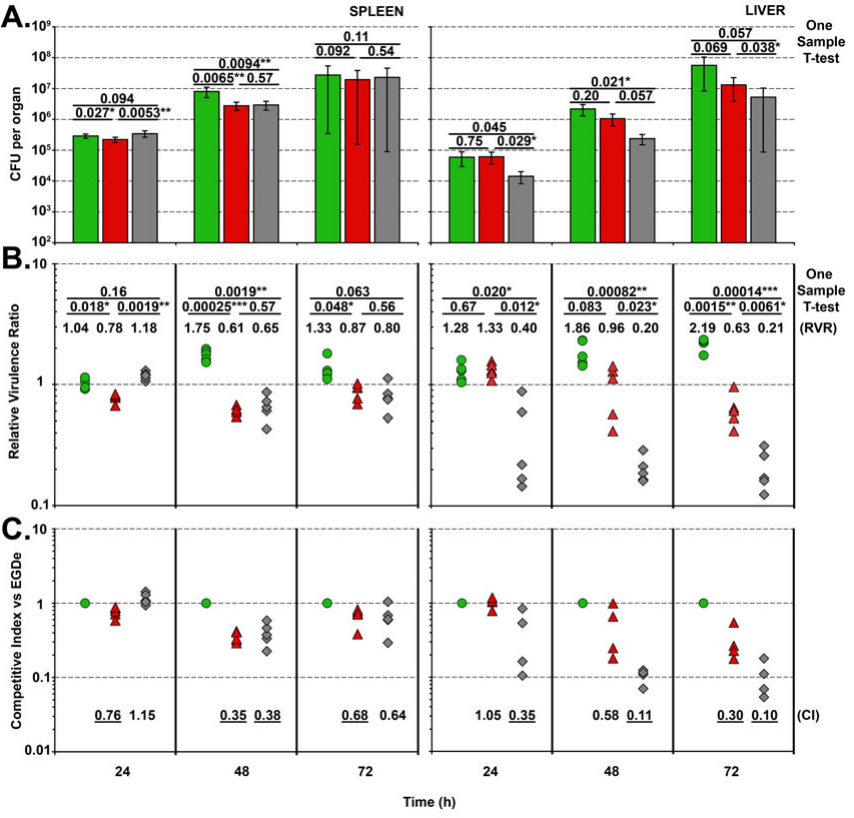
**Growth kinetics within Balb/c mice after IV infection with three *L. monocytogenes *strains**. (A) A tail vein injection containing an equal ratio of three *L. monocytogenes *strains (EGDe, 10403S and F2365) was administered into 15 Balb/c mice with a total inoculum of 2 × 10^4 ^CFU per 100 μl. Each of the strains was tagged with a different antibiotic marker. On each subsequent day, 5 mice were sacrificed, with the spleens and livers enumerated on different BHI agars as described in the materials and methods. Bars correspond to; green (EGDe::pIMC3*kan*), red (10403S::pIMC3*ery*) and grey (F2365::pIMC3*tet*). Data are presented as mean CFU per organ with standard deviation. Statistical analyses were conducted using the one sample T-test (see additional file 1) measuring the raw CFU differences between two strains to calculate the mean difference per organ over 5 mice. The relative virulence ratio (RVR) (B) was calculated from the data presented in (A) as described in figure legend 2. Symbols correspond to; green circle (EGDe::pIMC3*kan*), red diamond (10403S::pIMC3*ery*) and grey triangle (F2365::pIMC3*tet*). Statistical analyses were conducted using the one sample T-test measuring the RVR differences between two strains to calculate the mean difference of 5 organs (see additional file 2). The calculated P-values are presented, with values below 0.05 considered significant (P < 0.05 (*), P < 0.005 (**) and P < 0.0005(***)). (C) The competitive index score relative to EGDe (set as 1) was calculated by dividing the percentage of the strain (10403S or F2365) within an organ by the percentage value obtained for EGDe. The CI score per organ were plotted (with the same symbols as in (B)) and the mean CI written below. A CI score of 1 denotes no difference in the virulence compared to EGDe (for CI calaulation see additional figure 3). Underlined scores denote were statistical significance from (B) was observed.

Finally, we tested three genome-sequenced *L. monocytogenes *strains in a 1:1:1 infection; namely EGDe (Kan^R^) 10403S (Ery^R^) and F2365 (Tet^R^), to examine the kinetics of the individual strain infection and obtain information on the relative virulence of these commonly used laboratory strains. We observed differences in the multiplication of each strain within the organs analysed, with counts of EGDe predominating in both the spleen and liver over the other two strains (Fig 3a). By day 3 in the liver, EGDe out-competed 10403S, while 10403S was recovered in significantly higher levels than F2365. In almost all cases, the relative virulence ratio method (RVR) resulted in greatly improved statistical validity when compared to the use of raw CFU data (Fig 3b). This was especially true for organ counts after 72 hours, where a large degree of variation in the infection of livers and spleens was observed. However, when relative ratios were examined, the increased statistical robustness of the data resulted in easier identification of significant differences. The RVR can only be calculated when strains are mixed within a single mouse and relative ratios are calculated. An absolute value for the ability to compete is presented in Fig 3c, as the competitive index of each strain relative to EGDe. Here we show that in the spleen after 48 h, both 10403S and F2365 exhibit significantly reduced loads when compared to EGDe, with this margin narrowed at 72 h. In the livers, F2365 was significantly impaired throughout the three day time course compared to both EGDe and 10403S. Taken together, these results show that the number of bacteria present in the organs at 72 h can be ranked as follows; spleen – EGDe > 10403S = F2365 and liver – EGDe > 10403S > F2365.

## Discussion

Our prior experience with the pPL2 plasmid [15,16] indicated that the chloramphenicol resistance expressed was relatively low, therefore we were unable to directly select *L. monocytogenes *out of complex samples. This could be due to the inducible nature of gram-positive *cat *genes as described by Lovett [12]. To circumvent the requirement for induction of *cat *expression, we remodelled pPL2 to decrease plasmid size and exchange the inducible *cat *promoter for a highly expressed constitutive version. This increased the MIC of the new vector, pIMC, ten-fold compared to pPL2. The improved selection allows the isolation of tagged *L. monocytogenes *from complex environments such as intestinal contents or food samples. Further development of pIMC involved the creation of a suite of four vectors with diverse antibiotic and phenotypic markers to allow the discrimination of individual *L. monocytogenes *isolates. These were used to establish a competitive index assay to compare the relative fitness of *L. monocytogenes *strains. The tags did not compromise the fitness of the transformed strains as determined by growth in complex media and by the maintenance of an initial infection ratio through out a three day time course in Balb/c mice. The method established allows the direct discrimination of strains without post-enumeration manual scoring, such as PCR or replica plating [17,18]. The introduction of post enumeration scoring can introduce bias in the discrimination of bacteria, as only a small sample size is processed per organ. When samples can be directly enumerated, larger numbers can be analysed and the introduction of human error reduced. Also, the IPTG inducible promoter should help to decrease any potential consequences of marker expression during competitive growth.

We also observed improved statistical robustness when analysing data from competitively infected mice. We found that ratios were maintained throughout the course of infection (Fig 2b), suggesting that, as observed with *Salmonella typhimurium *[1], true replication of the inoculum occurs rather than clonal expansion. The statistical significance was especially evident when data from the third day post infection was analysed (Fig 3a). Large differences in the CFU counts isolated from individual livers and spleens were observed due to inherent variation in the infection process and animal-animal variability. However, analysis of the ratio of strains demonstrated improved statistical validity (Fig 3b). For the experiment reported in Fig 3, 15 mice were used, whereas traditionally 45 mice would have been required if each strain were analysed individually. Therefore, using mixed infections, fewer mice are required to produce better results, a powerful demonstration of the 'reduce and refine' principles.

Competitive index assays for *L. monocytogenes *have previously been described by two research groups. Auerbuch et al [18] established a strain of *L. monocytogenes *(10403S) tagged with Tn917 (Ery^R^), which mimics wild type 10403S infections. This has been widely applied by researchers using the 10403S strain [19-22]. However, the assay developed is only applicable to 10403S, requires further analysis post enumeration (i.e. patching of colonies) and only allows the comparison of wild type to a second unmarked strain. In 2006, a fluorescence based method for the discrimination of *L. monocytogenes *strains was established in a replicative plasmid system [23]. A total of five fluorescent proteins were shown to be functional, with three suitable for simultaneous use to distinguish strains (due to emission spectra). While the tagging method is non-invasive, prolonged incubation is required for chromophore maturation and in the absence of selective pressure, significant and variable plasmid loss was observed, depending on the fluorescent marker used [23]. The authors also describe difficulty in discriminating large virulence attenuations due to the non-selective nature of the marker.

From the comparison of three commonly used laboratory strains of *L. monocytogenes*, we observed distinct differences in the kinetics of infection. Upon re-examination of the sequence of the *inlB *gene from F2365, it was shown to encode a nonsense mutation leading to premature stop codon in the gene [24]. This may help to explain the impaired ability to establish infection within the liver compared to the 10403S and EGDe, as InlB plays an important role in liver colonization [8].

We have applied the competitive index experiments to in *vivo *experiments, but it equally could be used *in vitro *to examine growth or survival within both sterile and complex systems. Additionally, we envisage the use of internal controls in experiments, such as the comparison of mutant, wild type and complemented mutant, or the dual tagging of a wild type and two separate mutants in murine or tissue culture assays (e.g. adhesion, invasion and/or intracellular multiplication). We acknowledge that problems may be encountered in certain situations when comparing multiple strains in a single host, e.g. if a molecule involved in quorum sensing complemented a virulence defect or when the route of infection can be hijacked by a defective competing strain [25]. Nonetheless, we believe our protocol displays significant advantages over previously described methods and provides an additional tool for use in infection biology.

## Conclusion

We have established a method for the comparison of the relative fitness of *L. monocytogenes *strains utilising a novel, stable integrative plasmid, pIMC, based on the pPL2 phage integrase plasmid. This method allows for the selection and discrimination of up to four isolates from complex samples, without the requirement for post-enumeration processing. There were no detrimental effects on virulence or growth rate of the *L. monocytogenes *strains due to tag expression, as demonstrated by systemic spread in a BALB/c mouse model similar to that seen with a wild type strain. The utility of the method was demonstrated through the direct comparison of virulence of three commonly used *L. monocytogenes *strains (EGDe, 10403S and F2365). The data yielded improved statistical robustness when the ratios were examined rather than the cumulative CFU counts from spleens and livers. EGDe was shown to predominate in the liver and spleen by day three of infection compared to the other two strains. F2365 exhibited similar kinetics to 10403S in spleen replication but was significantly impaired in the ability to replicate within the liver when compared to both strains.

## Methods

### Strains, Antibiotics and Reagents

The bacterial strains, plasmids and oligonucleotides used in this study are described in table 1. *Escherichia coli *and *Listeria monocytogenes *were made electrocompetent using the protocols of Sheng et al [26] and Monk et al [27] respectively. *E. coli *was routinely cultured in Luria Bertani broth and *L. monocytogenes *in brain heart infusion broth (Oxoid) with shaking at 200 rpm. Broth media were solidified with 1.5% agar (Merck). For antibiotic selection the following concentrations (μg/ml) were used (*E. coli*-Ec, *L. monocytogenes*-Lm): Erythromycin (Ery) (Ec) 250 (Lm) 5, Chloramphenicol (Cm) (Ec) 10 (Lm) 7.5, Kanamycin (Kan) (Ec/Lm) 50 and Tetracycline (Tet) (Ec/Lm) 10. For the detection of beta-glucuronidase (Gus) activity, 5-Bromo-4-chloro-3-indolyl-beta-D-glucuronic Acid, Cyclohexylammonium Salt (X-gluc)(Calbiochem, Merck) was dissolved in Dimethylformamide at 40 mg/ml and was added to agar plates at 100 μg/ml. For the induction of marker expression, IPTG was included in agar at a concentration of 1 mM (Calbiochem, Merck). Colony (2× Master Mix, Promega) and high fidelity PCR (KOD Hotstart DNA polymease, Merck) was performed as described by Monk et al [27]. All reagents were purchased from Sigma Aldrich unless otherwise stated.

**Table 1:** Plasmids, Strains and Oligonucleotides

	**PLASMID **	
**Name**	**Description**	**Reference**
pTV1-OK	Temperature sensitive plasmid for the delivery of Tn917. 11 kb. Ery^R ^Kan^R^.	[33]
pNZ272	Lactococcal beta-glucuronidase (*gusA*) transcriptional fusion vector. 4.6 kb. Cm^R^.	[34]
pIMC	Site-specific Listerial integrative vector. High-level Cm^R ^from Phelp driven expression. 4.6 kb. Cm^R^.	This study
pIMK3	Site-specific Listerial integrative vector. High level IPTG controlled gene expression. 7.5 kb. Kan^R^.	[27]
pIMC3*kan*	Site-specific Listerial integrative vector. IPTG controlled expression of *aphA*-III ex pTV1-OK. Cm^R^	[33] This study
pIMC3*gus*	Site-specific Listerial integrative vector. IPTG controlled expression of *gusA *ex pNZ272. Cm^R^	[34] This study
pIMC3*ery*	Site-specific Listerial integrative vector. IPTG controlled expression of *ermAM *ex pTV1-OK. Cm^R^	[33] This study
pIMC3*tet*	Site-specific Listerial integrative vector. IPTG controlled expression of *tetM *ex CH919. Cm^R^	[35] This study

	**BACTERIA**	
***Listeria monocytogenes***		

EGDe	Wild type 1/2a strain. Genome sequenced.	[9]
10403S	Wild type 1/2a strain. Genome sequenced. Broad Institute (MIT).	[36]
F2365	Wild type 4b strain. Genome sequenced.	[10]
EGDe::pIMC3*kan*	EGDe transformed with pIMC3*kan *integrated at the tRNA^ARG^.	This study
EGDe::pIMC3*gus*	EGDe transformed with pIMC3*gus *integrated at the tRNA^ARG^.	This study
EGDe::pIMC3*ery*	EGDe transformed with pIMC3*ery *integrated at the tRNA^ARG^.	This study
EGDe::pIMC3*tet*	EGDe transformed with pIMC3*tet *integrated at the tRNA^ARG^.	This study
10403S::pIMC3*ery*	10403S transformed with pIMC3*ery *integrated at the tRNA^ARG^.	This study
F2365::pIMC3*tet*	F2365 transformed with pIMC*tet *integrated at the tRNA^ARG^.	This study
**Other Bacteria**		
DH10B	Wild type *Escherichia coli*. Used for routine cloning.	Invitrogen
CH919	MG1363 *Lactococcus lactis *subsp. *lactis *containing Tn919.	[36]

	**OLIGONUCLEOTIDE**	
**Name**	**5'-3'**^a^	**RE site**

108 Phelp B REV	CATGGGTTTCACTCTCCTTCTAC	
181 CAT C FWD	AAATGTAGAAGGAGAGTGAAACCCATGAACTTTAATAAAATTGATTTAGAC	
203 MCS FWD	AATTAACCCTCACTAAAGGGAAC	
204 MCS REV	GACGTCGTAATACGACTCACTATAGGGC	
205 Back FWD	AGTGAGTCGTATTACGACGTCCCAGGGCTTCCCGGTATCAAC	
206 Phelp A FWD	ATCCCATTATGCTTTGGCAGTTTATTC	
209 CAT D REV	ATAATGAGACAGAATTATGATGATCATATGTCAACTAACGGGGCAGG	
211 Back REV	CATATGATCATCATAATTCTGTCTCATTATATAAC	
373 *gusA *FWD	TATA**CCATGG**TACGTCCTGTAGAAACCCCAACC	*Nco*I
374 *gusA *REV	ATAT**CTGCAG**TTATTGTTTGCCTCCCTGCTGCGG	*Pst*I
385 *aphA3 *FWD	TAAT**CCATGG**CTAAAATGAGAATATCACCGGAATTG	*Nco*I
386 *aphA3 *REV	AAGC**CTGCAG**TTAAAGCTTTTTAGACATCTAAATCTAGG	*Pst*I
387 *ermAM *FWD	ATAT**CCATGG**ACAAAAATATAAAATATTCTCAAAAC	*Nco*I
388 *ermAM *REV	ATAT**CTGCAG**TTATTTCCTCCCGTTAAATAATAGATAAC	*Pst*I
408 *tetM *FWD	ATAT**CCATGG**AAATTATTAATATTGGAGTTTTAGC	*Nco*I
409 *tetM *REV	ATAT**CTGCAG**TTAAGTTATTTTATTGAACATATATCG	*Pst*I

^a ^Restriction sites are in bold type

### Construction of pIMC: a site specific integrative plasmid

The site-specific integrative listeriophage vector, pPL2, was modified by spliced overlap extension (SOE) PCR to incorporate constitutive chloramphenicol selection and decrease vector size. Initially three fragments were amplified: (A) pBluescriptII KS (+) multiple cloning site (MCS) including T3 and T7 promoter primer binding sites (IM203/204) (B) the pPL2 backbone from 1100 to 4585 ([GenBank:AJ417449]) (IM205/211) and (C) the Phelp promoter (from pPL2*lux*-Phelp) [13] fused to chloramphenicol acetyltransferase (*cat*) gene from pNZ8048 [28] by SOE PCR (IM181/209 and IM206/108). Phelp is a chimeric construct containing an optimized consensus lactococcal promoter P_CP25 _[29] joined to the 5' UTR of the *L. monocytogenes hlyA *gene [30]. The MCS of pBluescriptII KS (+) was joined to the backbone (1100 bp end) of pPL2 to remove both *cat *genes. The Phelp-*cat *amplimer was joined to the backbone (4585 end) of the previous PCR product. The PCR product for pIMC (4581 bp) was gel extracted and phosphorylated with T4 polynucleotide kinase (New England Biolabs). The product was ligated with LigaFAST rapid DNA ligation system (Promega) for 30 min at 22°C, transformed into DH10B with selection on LBA containing Cm at 10 μg/ml. A single clone of pIMC was fully sequenced and deposited under the accession number: [EMBL:AM940001]. The plasmid schematic (Fig 1a) was drawn with the online plasmid drawing tool [31] and exported as an .svg file on an iBook (Apple) from the Opera web browser into Adobe Illustrator CS2 (Adobe) for further manipulation as an .svg file.

### Competitive index assay constructs

The pIMK3 is a kanamycin version of pIMC [27] which contains the gram-positive functional *rrnB *transcription termination sequence up stream (*Aat*II/S*ac*I) of an IPTG inducible promoter (*Sac*I/*Nco*I). Located downstream of the IPTG inducible promoter is the highly expressed repressor, Phelp-*lacI *(*Sal*I/*Kpn*I). The above *Aat*II/*Kpn*I region was subcloned from pIMK3 into pIMC, producing pIMCa. The genes for antibiotic markers *tetM *from CH919 genomic DNA [32], *ermAM *from pTV1-OK [33], *aphA3 *from pTV1-OK [34] and the phenotypic marker *gusA *from pNZ272 [35] were cloned as *Nco*I/*Pst*I fragments into pIMK3 and confirmed phenotypically in *E. coli*. The marker and promoter were then subcloned as *Sac*I/*Pst*I fragments into the corresponding sites of pIMCa, yielding pIMC3*ery*, pIMC3*gus*, pIMC3*kan *and pIMC3*tet*. Constructs were transformed into *L. monocytogenes *generating strains: EGDe::pIMC3*ery*, EGDe::pIMC3*gus*, EGDe::pIMC3*kan*, EGDe::pIMC3*tet*, 10403S::pIMC3*ery *and F2365::pIMC3*tet*. The production of these constructs took advantage of the high-level CAT expression from pIMC to facilitate *in vivo *selection (from faecal/intestinal samples) and allow the discrimination of genotypes by inducible marker expression, when used in co-culture experiments.

### Competitive index assay experiments

Overnight cultures of the tagged strains were washed three times with PBS and diluted back to an OD_600 nm _of 0.1 (1× 10^8 ^cfu/ml) (Biophotometer, Eppendorf). Cultures were then subsequently mixed at ratios of 1:1:1:1, 83.3:13.2:2.75:0.75. (EGDe::pIMC3*gus*, EGDe::pIMC3*tet*, EGDe::pIMC3*ery *and EGDe::pIMC3*kan*, respectively) or 1:1:1 (EGDe::pIMC3*kan*, 10403S::pIMC3*ery *and F2365::pIMC3*tet*, respectively) and diluted 1:500 to obtain 2 × 10^5 ^cfu/ml. Fifteen specific-pathogen free female BALB/c mice (aged 6–8 weeks; Harlan) were intravenously inoculated via tail vein with 100 μl of diluted cells. The inoculum was enumerated on the following BHI based agars (A) BHI only (B) Cm 7.5 + 1 mM IPTG + X-gluc (C) Cm 7.5 + 1 mM IPTG + Kan (D) Cm 7.5 + 1 mM IPTG + Erm (E) Cm 7.5 + 1 mM IPTG + Tet. For the comparison of EGDe, 10403S and F2365, X-gluc containing agars were omitted. In preliminary experiments the incorporation of IPTG, X-gluc and Cm was shown not to impact on the recovery of *L. monocytogenes *from organ homogenates (data not shown). On days 1, 2 and 3 post infection, five mice from each group were sacrificed and spleens and livers aseptically removed. The organs were homogenized in 5 ml of PBS, serially diluted and enumerated on the agars described above. For the comparison of EGDe, 10403S and F2365 agar (B) was omitted.

The comparison of the tagged strains' ability to compete within a mouse was determined as follows: the CFU per organ from each strain within a single mouse was divided by the cumulative total of the four strains (addition of each single count) to obtain a ratio of each strain per organ. This ratio was divided by the initial ratio of the inoculum to obtain a relative virulence ratio (RVR). A RVR score of 1 denotes no change the ability to compete, e.g. for the comparison of EGDe, 10403S and F2365 the RVR scores were represented relative to EGDe (set as 1), thus obtaining a competitive index (CI) value. All procedures involving animals complied with relevant legislation and were approved by the animal ethics committee at University College Cork.

Statistical analysis (One sample T-test, Microsoft Excel) was applied to the raw CFU and RVR counts through the calculation of CFU or ratio difference (e.g. strain 1 to strain 2, strain 1 to strain 3 and strain 2 to strain 3) per organ (n = 5). A Microsoft Excel spreadsheet is attached for the calculation of the RVR and subsequent one sample T-test of CFU and RVR (additional files 1, 2 and 3). Competitive index was calculated relative to EGDe with the RVR of 10403S and F2365 per organ divided by the RVR of EGDe (additional file 3). The average CI from 5 organs is presented in Fig 3c.

## Authors' contributions

IRM conceived the study, carried out the main experimental work and drafted the manuscript. PGC carried out murine inoculations, dissection and helped draft the manuscript. MC performed statistical analyses on all data from the study. CGMG participated in the design and coordination of the study and helped to draft the manuscript. CH helped to conceive the study, interpret the data and draft the manuscript. All authors have read and approved the final manuscript.

## Supplementary Material

Additional file 1 Statistical analysis (One Sample T-test) of raw CFU. Attached is a Microsoft Excel spreadsheet for the statistical analysis from organ counts (e.g. spleen and liver). The raw CFU count from each strain per organ is inserted in the indicated boxes and the statistical analysis output is on the right hand side. Click here for file

Additional file 2 Calculation and statistical analysis (One Sample T-test) of the relative virulence ratio (RVR). Attached is a Microsoft Excel spreadsheet for the calculation and statistical analysis from organ counts (E.g. spleen and liver) converted into RVR. The raw CFU count from each strain per organ is inserted in the indicated boxes and the statistical analysis output from the RVR is on the right hand side. Click here for file

Additional file 3 Conversion of CFU to competitive index relative to strain 1. Attached is a Microsoft Excel spreadsheet for the conversion of organ counts (E.g. spleen and liver) to the RVR and subsequent calculation of competitive index relative to strain 1. The raw CFU count from each strain per organ is inserted in the indicated boxes and the CI calculated on the right hand side. Click here for file

## References

[B1] Beuzon CR, Holden DW (2001). Use of mixed infections with Salmonella strains to study virulence genes and their interactions in vivo. Microbes Infect.

[B2] Falkow S (2004). Molecular Koch's postulates applied to bacterial pathogenicity–a personal recollection 15 years later. Nat Rev Microbiol.

[B3] Lenz LL, Mohammadi S, Geissler A, Portnoy DA (2003). SecA2-dependent secretion of autolytic enzymes promotes *Listeria monocytogenes *pathogenesis. Proc Natl Acad Sci USA.

[B4] Lowe BA, Miller JD, Neely MN (2007). Analysis of the polysaccharide capsule of the systemic pathogen *Streptococcus iniae *and its implications in virulence. Infect Immun.

[B5] Pratt JT, Tamayo R, Tischler AD, Camilli A (2007). PilZ domain proteins bind cyclic diguanylate and regulate diverse processes in *Vibrio cholerae*. J Biol Chem.

[B6] Vazquez-Boland JA, Kuhn M, Berche P, Chakraborty T, Dominguez-Bernal G, Goebel W, Gonzalez-Zorn B, Wehland J, Kreft J (2001). Listeria pathogenesis and molecular virulence determinants. Clin Microbiol Rev.

[B7] Mengaud J, Ohayon H, Gounon P, Mege R-M, Cossart P (1996). E-cadherin is the receptor for internalin, a surface protein required for entry of *L. monocytogenes *into epithelial cells. Cell.

[B8] Khelef N, Lecuit M, Bierne H, Cossart P (2006). Species specificity of the *Listeria monocytogenes *InlB protein. Cell Microbiol.

[B9] Glaser P, Frangeul L, Buchrieser C, Rusniok C, Amend A, Baquero F, Berche P, Bloecker H, Brandt P, Chakraborty T, Charbit A, Chetouani F, Couve E, de Daruvar A, Dehoux P, Domann E, Dominguez-Bernal G, Duchaud E, Durant L, Dussurget O, Entian KD, Fsihi H, Garcia-del Portillo F, Garrido P, Gautier L, Goebel W, Gomez-Lopez N, Hain T, Hauf J, Jackson D, Jones LM, Kaerst U, Kreft J, Kuhn M, Kunst F, Kurapkat G, Madueno E, Maitournam A, Vicente JM, Ng E, Nedjari H, Nordsiek G, Novella S, de Pablos B, Perez-Diaz JC, Purcell R, Remmel B, Rose M, Schlueter T, Simoes N, Tierrez A, Vazquez-Boland JA, Voss H, Wehland J, Cossart P (2001). Comparative genomics of Listeria species. Science.

[B10] Nelson KE, Fouts DE, Mongodin EF, Ravel J, DeBoy RT, Kolonay JF, Rasko DA, Angiuoli SV, Gill SR, Paulsen IT, Peterson J, White O, Nelson WC, Nierman W, Beanan MJ, Brinkac LM, Daugherty SC, Dodson RJ, Durkin AS, Madupu R, Haft DH, Selengut J, Van Aken S, Khouri H, Fedorova N, Forberger H, Tran B, Kathariou S, Wonderling LD, Uhlich GA, Bayles DO, Luchansky JB, Fraser CM (2004). Whole genome comparisons of serotype 4b and 1/2a strains of the food-borne pathogen *Listeria monocytogenes *reveal new insights into the core genome components of this species. Nucleic Acids Res.

[B11] Lauer P, Chow MY, Loessner MJ, Portnoy DA, Calendar R (2002). Construction, characterization, and use of two *Listeria monocytogenes *site-specific phage integration vectors. J Bacteriol.

[B12] Lovett PS (1996). Translation attenuation regulation of chloramphenicol resistance in bacteria–a review. Gene.

[B13] Riedel CU, Monk IR, Casey PG, Morrissey D, O'Sullivan GC, Tangney M, Hill C, Gahan CG (2007). Improved luciferase tagging system for *Listeria monocytogenes *allows real-time monitoring in vivo and in vitro. Appl Environ Microbiol.

[B14] Camilli A, Tilney LG, Portnoy DA (1993). Dual roles of plcA in *Listeria monocytogenes *pathogenesis. Mol Microbiol.

[B15] Monk IR, Cook GM, Monk BC, Bremer PJ (2004). Morphotypic conversion in *Listeria monocytogenes *biofilm formation: biological significance of rough colony isolates. Appl Environ Microbiol.

[B16] Bron PA, Monk IR, Corr SC, Hill C, Gahan CG (2006). Novel luciferase reporter system for in vitro and organ-specific monitoring of differential gene expression in *Listeria monocytogenes*. Appl Environ Microbiol.

[B17] Sjostrom K, Blomberg C, Fernebro J, Dagerhamn J, Morfeldt E, Barocchi MA, Browall S, Moschioni M, Andersson M, Henriques F, Albiger B, Rappuoli R, Normark S, Henriques-Normark B (2007). Clonal success of piliated penicillin nonsusceptible pneumococci. Proc Natl Acad Sci USA.

[B18] Auerbuch V, Lenz LL, Portnoy DA (2001). Development of a competitive index assay to evaluate the virulence of *Listeria monocytogenes *actA mutants during primary and secondary infection of mice. Infect Immun.

[B19] Nadon CA, Bowen BM, Wiedmann M, Boor KJ (2002). Sigma B contributes to PrfA-mediated virulence in *Listeria monocytogenes*. Infect Immun.

[B20] Xu H, Chun T, Choi HJ, Wang B, Wang CR (2006). Impaired response to Listeria in H2-M3-deficient mice reveals a nonredundant role of MHC class Ib-specific T cells in host defense. J Exp Med.

[B21] D'Orazio SE, Velasquez M, Roan NR, Naveiras-Torres O, Starnbach MN (2003). The *Listeria monocytogenes *lemA gene product is not required for intracellular infection or to activate fMIGWII-specific T cells. Infect Immun.

[B22] O'Neil HS, Marquis H (2006). *Listeria monocytogenes *flagella are used for motility, not as adhesins, to increase host cell invasion. Infect Immun.

[B23] Andersen JB, Roldgaard BB, Lindner AB, Christensen BB, Licht TR (2006). Construction of a multiple fluorescence labelling system for use in co-invasion studies of *Listeria monocytogenes*. BMC Microbiol.

[B24] Nightingale KK, Milillo SR, Ivy RA, Ho AJ, Oliver HF, Wiedmann M (2007). *Listeria monocytogenes *F2365 carries several authentic mutations potentially leading to truncated gene products, including inlB, and demonstrates atypical phenotypic characteristics. J Food Prot.

[B25] Ginocchio C, Pace J, Galan JE (1992). Identification and molecular characterization of a *Salmonella typhimurium *gene involved in triggering the internalization of salmonellae into cultured epithelial cells. Proc Natl Acad Sci USA.

[B26] Sheng Y, Mancino V, Birren B (1995). Transformation of *Escherichia coli *with large DNA molecules by electroporation. Nucleic Acids Res.

[B27] Monk IR, Gahan CG, Hill C (2008). Tools for functional post-genomic analysis of *Listeria monocytogenes*. Appl Environ Microbiol.

[B28] Mierau I, Kleerebezem M (2005). 10 years of the nisin-controlled gene expression system (NICE) in *Lactococcus lactis*. Appl Microbiol Biotechnol.

[B29] Jensen PR, Hammer K (1998). The sequence of spacers between the consensus sequences modulates the strength of prokaryotic promoters. Appl Environ Microbiol.

[B30] Shen A, Higgins DE (2005). The 5' untranslated region-mediated enhancement of intracellular listeriolysin O production is required for *Listeria monocytogenes *pathogenicity. Mol Microbiol.

[B31] Scalable Vector graphics: plasmid map. http://bioinformatics.org/savvy/.

[B32] Hill C, Venema G, Daly C, Fitzgerald GF (1988). Cloning and characterization of the tetracycline resistance determinant of and several promoters from within the conjugative transposon Tn919. Appl Environ Microbiol.

[B33] Brehm J, Salmond G, Minton N (1987). Sequence of the adenine methylase gene of the *Streptococcus faecalis *plasmid pAM beta 1. Nucleic Acids Res.

[B34] Gutierrez JA, Crowley PJ, Brown DP, Hillman JD, Youngman P, Bleiweis AS (1996). Insertional mutagenesis and recovery of interrupted genes of *Streptococcus mutans *by using transposon Tn917: preliminary characterization of mutants displaying acid sensitivity and nutritional requirements. J Bacteriol.

[B35] Platteeuw C, Simons G, de Vos WM (1994). Use of the Escherichia coli beta-glucuronidase (gusA) gene as a reporter gene for analyzing promoters in lactic acid bacteria. Appl Environ Microbiol.

[B36] Hill C, Daly C, Fitzgerald GF (1987). Development of High-Frequency Delivery System for Transposon Tn919 in Lactic Streptococci: Random Insertion in *Streptococcus lactis *subsp. *diacetylactis *18–16. Appl Environ Microbiol.

[B37] Bishop DK, Hinrichs DJ (1987). Adoptive transfer of immunity to *Listeria monocytogenes*. The influence of in vitro stimulation on lymphocyte subset requirements. J Immunol.

